# Three Novel C-Repeat Binding Factor Genes of *Dimocarpus longan* Regulate Cold Stress Response in Arabidopsis

**DOI:** 10.3389/fpls.2020.01026

**Published:** 2020-07-07

**Authors:** Xiaoyan Yang, Rui Wang, Haohao Jing, Qiuyu Chen, Xiuli Bao, Jietang Zhao, Guibing Hu, Chengming Liu, Jiaxin Fu

**Affiliations:** ^1^Key Laboratory of Biology and Genetic Improvement of Horticultural Crops (South China), Ministry of Agriculture and Rural Affairs/Guangdong Litchi Engineering Research Center, College of Horticulture, South China Agricultural University, Guangzhou, China; ^2^College of Horticulture and Gardening, Yangtze University, Jingzhou, China

**Keywords:** *Dimocarpus longan*, cold stress, *DlCBF1/2/3*, expression pattern, transgenic Arabidopsis

## Abstract

Longan (*Dimocarpus longan*) is a typical southern subtropical fruit tree species that is sensitive to cold stress. C-repeat binding factors (CBFs), as transcription factors, are crucial components involved in the molecular regulation of the plant response to cold stress. However, the role of CBF homologs in the cold response regulation of longan remains largely unknown. Here, three novel CBF genes, *DlCBF1*, *DlCBF2*, and *DlCBF3*, were cloned from longan. DlCBF1 and DlCBF2 contain an AP2 domain and PKKPAGR and DSAWR CBF signature motifs, while DlCBF3 has mutations within these conserved signature motifs. DlCBF1/2/3 were mainly localized in the nucleus and specifically bound to CRT/DRE cis-elements, resulting in strong transcriptional activation. *DlCBF1/2* exhibited tissue expression specificity, and their expression was induced by low temperature, while *DlCBF3* had no tissue specificity and barely responded to low temperature. *DlCBF1, DlCBF2*, and *DlCBF3* overexpression in Arabidopsis-enhanced cold tolerance by increasing proline accumulation and reducing reactive oxygen species (ROS) content, accompanied by upregulated expression of cold-responsive genes (*AtRD29A*, *AtCOR15A*, *AtCOR47*, and *AtKIN1*) in the CBF cold stress response signaling pathway. In conclusion, the biological functions of *DlCBF1/2/3* were somewhat conserved, but slow expression of *DlCBF1*/*2* and low expression of *DlCBF3* may partly cause the cold sensitivity of longan. Collectively, these results indicated that differences exist in the expression and function of CBF orthologs in the cold-sensitive plant species longan, and these findings may help to improve the understanding of the cold response regulation mechanism and provide important theoretical support for cold-tolerant breeding of longan.

## Introduction

Cold stress is an adverse abiotic factor that restricts the geographical distribution of plants and influences crop growth and development, resulting in decreased productivity and quality of many important crop species (Pearce, 2001). To cope with cold environments and survive, plants have evolved sophisticated physiological, biochemical, and molecular regulatory mechanisms to increase their cold tolerance (Thomashow, 1999; Kaplan et al., 2007). Accumulating evidence has revealed that C-repeat binding factor (CBF) transcription factors (TFs) play important roles in the stress response and in plant growth and development. Therefore, plants form a series of precise positive and negative regulatory networks to regulate the expression of CBF genes to maintain the dynamic balance between plant growth and environmental adaptation (Liu et al., 2018). Cold-induced CBF genes specifically recognize and bind to the conserved C-repeat/dehydration response motif (CRT/DRE, CCGAC), which is present in the promoters of genes, thereby inducing the expression of cold-regulated (*COR*) genes (Stockinger et al., 1997; Medina et al., 2011). *COR* genes encode cryoprotective proteins and some key enzymes involved in the accumulation of metabolites (osmolytes) that enhance the cold tolerance of plants (Ding et al., 2019).

CBFs are typical APETALA 2/Ethylene Responsive Factor (AP2/ERF) TFs. CBF proteins contain an AP2/ERF domain and PKKPAGR (PKKP/RAGRxKFxETRHP) and DSAWR CBF signature motifs (Jaglo et al., 2001). The expression of *CBF*s in many plant species can be induced by low temperature. In Arabidopsis, *AtCBF1/2/3* (*DREB1C/DREB1B/DREB1A*) genes are cold-induced CBF genes. The expression of these *AtCBF1/2/3* genes is specifically induced within 15 min and peaks within 3 h after cold treatment (Gilmour et al., 1998; Novillo et al., 2004; Medina et al., 2011). *AtCBF1/2/3* overexpression can promote *COR* expression and enhance the cold tolerance of transgenic Arabidopsis (Jaglo et al., 1998). Recent reports have revealed that *cbfs* triple mutants are remarkably sensitive to freezing temperature after cold acclimation; consistently, the expression of 10% to 20% of *COR* genes decreased in *cbfs* triple mutants under freezing stress (Jia et al., 2016; Zhao et al., 2016). These results indicated that the CBF-COR regulatory pathway is a core component involved in the molecular regulation of the cold response in plants.

CBF homologous genes have been characterized in various plant species, including *Zea mays* (Qin et al., 2004), *Oryza sativa* (Dubouzet et al., 2003; Ito et al., 2006), poplar (Benedict et al., 2006), citrus (Champ et al., 2007), and apple (Feng et al., 2012). Heterologous expression of *AtCBF1* in tomato enhances tolerance to cold and oxidative stresses (Hsieh et al., 2002). *AtCBF3* overexpression in cassava was shown to improve the cold and drought resistance of transgenic plants (An et al., 2016), and heterologous expression of Vitis *VvCBF1/4* and sweet cherry *PaCBF* in Arabidopsis promotes freezing tolerance in transgenic plants (Siddiqua and Nassuth, 2011). Overall, these studies indicate that the functions of CBFs are highly conserved in the regulation of cold responses among different plant species.

Accumulating studies on Arabidopsis indicate that the expression of CBF genes is regulated by the circadian clock and light signals. Circadian Clock-associated 1 (CCA1), Late Elongated Hypocotyl (LHY), and Pseudo Response Regulators (PRRs) are the core components of the circadian clock. Among of them, CCA1 and LHY active *CBF* expression and the downstream *COR* genes to positively regulate freezing tolerance, but PRR5/7/9 repress *CBF* expression and negatively regulates freezing tolerance (Nakamichi et al., 2009; Dong et al., 2011). Emerging evidence has shown that phytochrome-interacting factor 4/7 (PIF4/7) TFs repress *CBF* expression under long day (LD) conditions (Kidokoro et al., 2009; Lee and Thomashow, 2012). PIF3 repress *CBF* expression and negatively regulates freezing tolerance in dark conditions (Jiang et al., 2017) but interacts with CBF proteins and enhances freezing tolerance by stabilizing phytochrome B (phyB) in light conditions under cold stress (Jiang et al., 2020). In addition, the expression and function of CBF orthologs is different in some cold-sensitive plant species, such as *Oryza sativa* and tomato. *OsDREB1A/B* was induced and peaked within 5 to 10 h after cold treatment (Dubouzet et al., 2003). Tomato contains three CBF genes, *LeCBF1/2/3*, of which only *LeCBF1* was induced by cold stress, and *LeCBF1* overexpression increased the freezing resistance of tomato. Fewer CBF-regulated proteins are present in tomato than in Arabidopsis (Zhang et al., 2004). These studies revealed that the function of CBFs in cold response regulation was specific in different species.

Longan (*Dimocarpus longan* Lour.) is an important evergreen fruit tree species in the Sapindaceae family. Longan subtropical fruit are not only delicious but also an essential source of traditional Chinese medicines because of their rich contents of secondary metabolites such as phenols. As a commercial fruit crop species, longan is widely cultivated in tropical and subtropical regions of China and Southeast Asian countries (Mei et al., 2014). Nevertheless, longan, which originated in South China or Southeast Asia (Lin et al., 2017), is sensitive to cold. In recent years, unpredictable frost-inducing weather has been frequently occurring in southern China; consequently, longan fruit production and quality have been significantly affected by the chilling (0–5°C) temperature, and trees have even died in freezing (<0°C) climate conditions, resulting in severe economic losses in the longan industry. The effective solution is to develop cold-tolerant longan cultivars to reduce damage caused by cold stress. However, due to the high degree of genome heterozygosity and long juvenile period, little progress has been made in breeding cold-tolerant longan. Therefore, there is an urgent need to investigate the molecular response mechanisms to cold stress and promote the breeding of cold-resistant longan.

To date, few studies have examined the molecular mechanisms of the longan response to cold stress. In this study, three longan CBF genes, *DlCBF1*, *DlCBF2*, and *DlCBF3*, were isolated, and their biological functions in the cold stress response were characterized. The results revealed that the biological functions of *DlCBF1/2/3* in the longan response to cold stress are conserved to some extent but that the expression was insufficient, especially for *DlCBF3*, which might partly result in longan cold sensitivity.

## Materials and Methods

### Plant Materials and Treatments

The main longan (*D. longan* Lour.) cultivar “Shixia” was used as the material in this study. The tested 5-year-old trees were cultivated in the longan germplasm resource nursery of South China Agricultural University, Guangzhou (23.12°N, 113.35°E), China. During late December of 2016, the seasonal temperature decreased to 10°C/5°C (daytime/night). At 9:00 am on the coldest day of this year (24/12/2016, 7°C/2°C) (**Supplementary Figure 1**), samples of different tissues, including apical buds, young red leaves, mature autumn leaves, young annual stems, and petioles, were collected for tissue-specific expression analysis of *DlCBFs*. To reduce environmental and physiological influences, 2-year-old “Shixia” longan grafted plants were incubated in 25°C growth chambers for one month with a 16-h light/8-h dark photoperiod, 100 μmol/ms^2^ illumination intensity, and 60% relative humidity and then transferred to 4°C growth chambers with same illumination conditions at 9:00 am for cold treatment (Peng et al., 2016). The leaves were sampled at 0, 1, 3, 6, 12, and 24 h after treatment to analyze the expression of *DlCBFs* in response to cold stress. Shoots incubated in normal conditions (25°C) were used as controls. All samples were frozen immediately in liquid nitrogen and stored at −80°C for further analysis.

The wild-type (WT) *Arabidopsis thaliana* ecotype Columbia (Col-0) was used for genetic transformation in this study. Transgenic lines and WT plants were grown in pots filled with a 3:1 mixture of soil and vermiculite at 22°C in a greenhouse under a normal 16-h light/8-h dark photoperiod, 100 μmol/ms^2^ light intensity, and 60% relative humidity (Shi et al., 2012). To assess the freezing tolerance of the transgenic lines, 3-week-old T_3_ homozygous and WT seedlings were transferred to 4°C growth chambers with same ambient conditions for 48 h and then were exposed to −4°C for 6 h, followed by 12 h of darkness at 4°C, after which they were returned to normal conditions for recovery for 6 days. The plant survival rates and phenotypes were recorded. The rosette leaves were sampled at 9:00 am on the day of the 4°C cold treatment initiation for physiological indexes and for cold-responsive gene expression analysis.

### Gene Cloning and Sequence Analysis

Total RNA was extracted using an RNAprep Pure Plant Kit (Tiangen, China), and first-strand cDNA was synthesized using a PrimeScript™ II 1st Strand cDNA Synthesis Kit (Takara, Japan) according to the manufacturers' instructions. The putative *DlCBF* genes were obtained by BLAST analysis using TBtools (Chen et al., 2020) in conjunction with the longan genome database (Lin et al., 2017). The specific primers used for gene amplification are listed in **Supplementary Table 1**. Bioinformatic analysis of DlCBFs and multiple alignment of deduced amino acid sequences were conducted using ClustalX and GeneDoc software, respectively (Wang et al., 2018). A phylogenetic tree was constructed with MEGA 7.0 using the neighbor-joining (NJ) method, and bootstrap values with 1,000 replicates were used (Kumar et al., 2016).

### Gene Expression Analysis

Gene transcript levels were evaluated using real-time quantitative PCR (RT-qPCR) with an ABI 7500 Real-time PCR System (Life Technologies Corporation, Beverly, MA, USA) together with 2× SYBR Green Real MasterMix (SYBR Green, Applied Biosystems) according to the manufacturer's instructions. The 2^−ΔΔCt^ method was used to calculate the relative expression of the target genes between samples (Livak and Schmittgen, 2001). Expression of *DlCBFs* and the typical *COR* genes in the CBF cold signaling pathway such as *AtRD29A, AtCOR15A, AtCOR47*, and *AtKIN1* (Stockinger et al., 1997) were detected by RT-qPCR. Longan *β-actin* and *AtACTIN2* (AT1G13320) were used as internal controls for longan and Arabidopsis, respectively (Ding et al., 2015; Wu et al., 2016). The RT-qPCR primers used in this study are listed in **Supplementary Table 1**.

### Vector Construction

The *DlCBF* open reading frames (ORFs) without their stop codons were subcloned into pGreen-35S-GFP vectors for subcellular localization analysis (Jiang et al., 2019). The full-length coding regions of the *DlCBF*s were cloned into pGBKT7 and pPZP6k90 vectors for transcriptional activation assays and for Arabidopsis transformation, respectively (Yang et al., 2019). To test the CRT/DRE-binding specificity of the DlCBFs, full-length *DlCBF* sequences were inserted into pGreen II 62-SK vectors as effectors. The longan CRT/DRE motif repeats (3× G**CCGAC**AGG, 3× DlCRT/DRE) or mutant CRT/DRE sequence repeats (3× G**AATCA**AGG, 3× DlCRT/DREmt) (Liu et al., 1998) were inserted into a pGreen II 0800-LUC vector as reporters. Fusion plasmids were constructed using an In-Fusion™ PCR Cloning Kit (Clontech, USA) and verified by sequencing. The primers and restriction sites used for vector construction are listed in **Supplementary Table 2**.

### Subcellular Localization, Transcriptional Activation, and CRT/DRE−Binding Activity Assay of DlCBFs

The 35S:DlCBFs-GFP and 35S: GFP fusion plasmids the empty vector were introduced into *Agrobacterium tumefaciens* strain GV3101 (psoup-p19), which were subsequently infiltrated into the abaxial surfaces of *Nicotiana benthamiana* leaves for transient transformation (Sparkes et al., 2006). The GFP fluorescence signals of leaf protoplasts were detected with a fluorescence microscope (Olympus BX53) after incubation with 0.1 μg/ml 4′,6-diamidino-2-phenylindole (DAPI) for 15 min. The pGBKT7-DlCBFs and pGBKT7-p53 constructed plasmids and pGBKT7 empty plasmids were transferred into a Y2HGold yeast strain independently using the lithium acetate method (PT1172-1, Clontech). The transformed yeast cells were cultured on plates containing SD/-Trp and SD/-Trp-His-Ade media. The yeast cell growth status and the activity of α-galactosidase were observed after incubation with 20 μg/ml X-α-gal for 10 to 30 min. For CRT/DRE-binding specificity of DlCBF analysis, the pGreenII 62-SK-DlCBF, pGreenII 0800-3DlCRT-LUC and pGreenII 0800-3DlCRTmt-LUC fusion construct plasmids were transformed into *A. tumefaciens* strain GV3101 (psoup-p19). The effector and reporter were mixed at a volumetric ratio of 9:1 and then infiltrated into the abaxial surfaces of *N. benthamiana* leaves for transient transformation. After inoculation for 48 to 72 h, LUC and REN luciferase activities were measured using a Dual-Luciferase Reporter Assay System (E1910, Promega) as described previously (Fan et al., 2016). The CRT/DRE-binding activity of the DlCBFs was calculated by the ratio of LUC to REN. At least six independent replicates were included for each pair.

### Arabidopsis Transformation

The pPZP6k90-DlCBF recombinant constructs containing the coding regions for longan *CBF1*, *CBF2*, and *CBF3* were introduced into *A. tumefaciens* strain GV3101 and transformed into WT Arabidopsis Col-0 by the floral dip method (Clough and Bent, 1998). Independent transgenic lines were screened on Murashige and Skoog (MS) media supplemented with kanamycin (100 mg/L) and further verified by genomic PCR using the gene-specific primers for the *DlCBF*s listed in **Supplementary Table 1**. T_3_ homozygous transgenic lines were used for cold tolerance assessments and physiological index measurements.

### Measurements of Ion Leakage, Proline, Malondialdehyde, and Reactive Oxygen Species Contents

Ion leakage was determined as described previously (Ding et al., 2015). Proline accumulation was measured by the sulfosalicylic acid-ninhydrin method as described by (Shi et al., 2012), and malondialdehyde (MDA) contents were measured using the thiobarbituric acid method, as described previously (Guo and Crawford, 2005). Hydrogen peroxide (H_2_O_2_) and superoxide (O_2_^.−^) contents were quantified using specific detection kits (Suzhou Comin Biotechnology, China) according to the manufacturer's instructions. Details of the methods are available in the study by Yang et al. (2019).

### Data Analysis

Each experiment included three biological replicates. The data represent the means ± SDs, and significant differences between experimental data were evaluated by Duncan's multiple comparison tests.

## Results

### Cloning and Identification of *DlCBF1/2/3* Genes

Three homologous longan CBF genes named *DlCBF1*, *DlCBF2*, and *DlCBF3* were isolated from the longan genome. The *DlCBF1/2/3* genes were located on three different scaffolds and had no introns within coding regions of longan genomic DNA. The full-length coding DNA sequences (CDSs) of the *DlCBF1*/*2*/*3* genes were 687, 726, and 636 bp, encoding 228, 241, and 211 deduced amino acids with molecular weights of 25.13, 26.77, and 23.71 kDa and pIs of 5.21, 6.24, and 5.60, respectively. The details have been deposited in the GenBank database (GenBank accession nos. MN504651, MN504652, and MN504653) and are listed in **Supplementary Table 3**. Sequence comparison revealed that the similarity coefficient between the three *CBF*s ranged from 58.88% to 38.59% (**Supplementary Table 4**).

**Figure 1:**
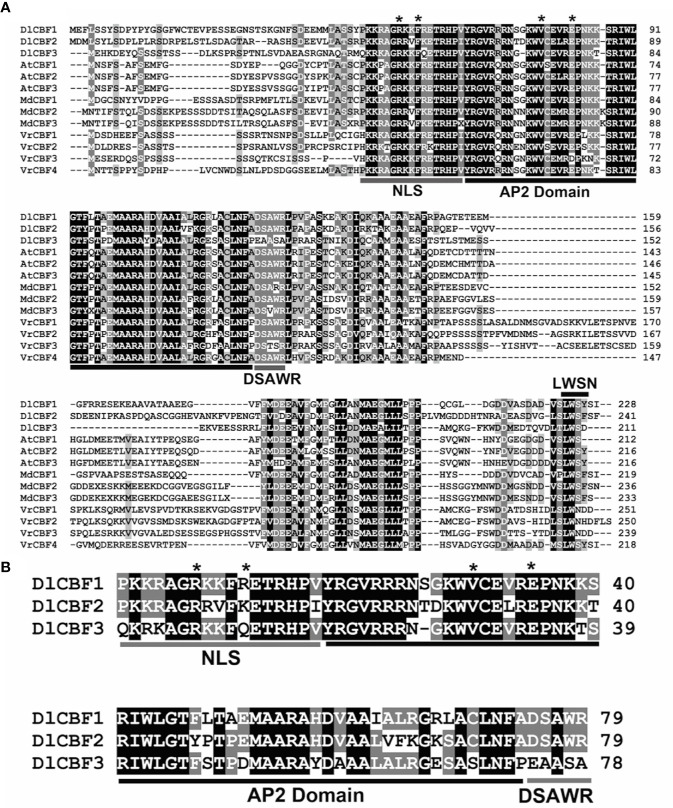
Amino acid sequence analysis of *DlCBF1/2/3*. **(A)** Multiple alignment of DlCBF1/2/3 and other CBF family proteins, including those of *D. longan* (DlCBF1/2/3, MN504651/MN504652/MN504653), Arabidopsis (AtCBF1/2/3, NP_567721.1/ABV27106.1/ABV27138.1), *Malus domestica* (MdCBF1/2/3, Z20446.1/AGM16327.1/AGL07697.1) and *V. riparia* (VrCBF1/2/3/4, R28671/R28674/R28675/W58104). The highly conserved amino acid residues are shaded black and grey. The predicted conserved motifs of the CBF proteins are labeled as an NLS domain (PKK/RPAGRxKFxETRHP), a DSAWR domain, an AP2 DNA-binding domain and an LWSY/F domain. The conserved sites are indicated by asterisks. **(B)** Sequence alignment of DlCBF1/2/3 conserved regions. Identical and similar amino acid residues are represented by black and gray shading, respectively. Conserved regions of the CBF proteins are labeled as an AP2 domain and as “CBF signature” (NLS and DSAWR) conserved sequences. The conserved amino acid sites are indicated by black asterisks.

**Figure 2:**
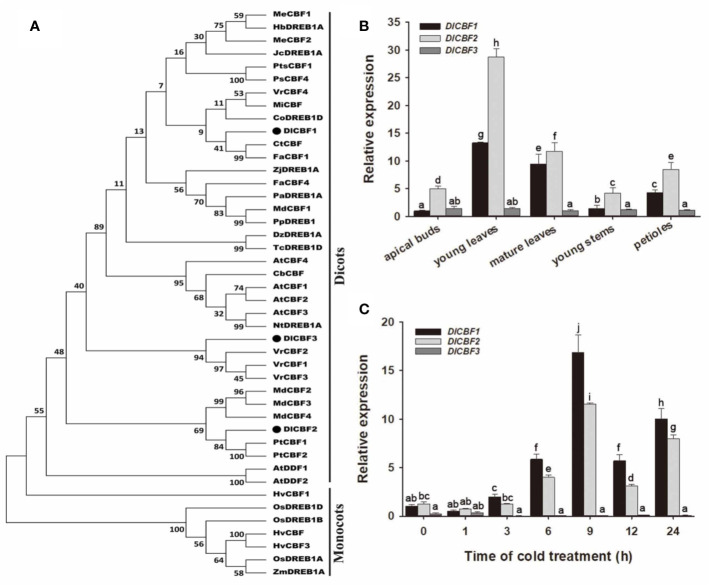
Phylogenetic and expression analyses of *DlCBF1/2/3*. **(A)** Phylogenetic analysis of DlCBF1/2/3 and homologs from different plant species. Accession IDs are as follows: CbCBF25, R35030; CpDREBP1D, XP_021903812; CpDREBP1D, XP_021903812; CtCBF, ABH08745.1; CaDREBP1D, AKF15923.1; DzDREBP1A, XP_022736454; FaCBF1/4, ABV65907.2/AEK94313; HbDREBP1A, XP_021649059.1; HvCBF1/3, AAL84170.1/AAX23692.1; HvCBF, AAG59618.1; JcDREBP1A, XP_012090326.1; JsDREBP1D, XP_018830708; MdCBF4, AGL07696.1; MiCBF, AIY26287.1; MeCBF1/2, AFA50331.1/AFA50332.1; NtDREB1A, ABD65969; OsDREB1A/1B/1D, AAN02486.1/AAX28958/AAX23721; PsCBF4, AIU92948.1; PtCBF1/2, ABO48363/ABC79627.1; PaDREBP1A, XP_021826871; PpDREB1, AEG64738; TcDREBP1D, XP_017973052; TcDREBP1D, XP_017973052; VrCBF1/2/3/4, R28671/R28674/R28675/W58104; ZmDR1B1A, AAN76804.1; and ZjDREBP1A, XP_015867545. **(B**, **C)** Tissue-specific expression and time course expression patterns of *DlCBF1/2/3* in response to cold stress. The relative expression of *DlCBF1* in apical buds and under normal temperature was used as a control. *DlACTIN* was used as an internal standard. The error bars indicate standard deviations (SDs) from three biological replicates. The different letters above bars indicate significant differences at the P < 0.05 level according to Duncan's multiple comparison tests.

Multiple sequence alignments showed that both DlCBF1 and DlCBF2 are typical CBF TFs in longan and contained an AP2/ERF domain and the nuclear localization signal (NLS, PKKP/RAGRxKFxETRHP) and DSAWR CBF signature sequences (Jaglo et al., 2001), as well as the LWSN conserved sequence within the C-terminus. However, the DlCBF3 amino acid sequences of the PKKP/R and DSAWR conserved domains were mutated to QKRK and EAASA, respectively (**Figures 1A, B**). Phylogenetic analysis showed that CBF proteins widely exist in dicotyledonous and monocotyledonous plants and are highly conserved in different plant species. DlCBF1/2/3 proteins are most closely related to the CtCBF protein of *Citrus trifoliata*, the PtCBF protein of *Populus trichocarpa* and the VrCBF protein of *Vitis riparia* (**Figure 2A**). CtCBF, PtCBF, and VrCBF have been reported to be involved in signal transduction as part of the response to cold (Benedict et al., 2006; Champ et al., 2007; Siddiqua and Nassuth, 2011). Therefore, it was speculated that DlCBF1/2/3 might play a similar role in the response to low temperature in longan.

### Tissue-Specific and Cold-Inducible Expression of *DlCBF1/2/3*

To understand the potential function of *DlCBF1/2/3* in the cold response of longan, we performed RT-qPCR to examine the expression patterns of *DlCBF1/2/3* in various tissues of Shixia during winter, including young leaves, mature leaves, apical buds, young stems, and petioles (**Figure 2B**). The *DlCBF1/2* genes were expressed the most in the young leaves and lowest in the apical buds, while the expression of *DlCBF3* was low in all the tested tissues. When the mature autumn leaves were treated with 4°C, the expression of *DlCBF1/2* was significantly induced at 3 h, gradually increased to the peak value at 9 h, and then decreased. However, the expression of *DlCBF3* was consistently low, and the expression trend did not change significantly (**Figure 2C**). These results suggested that the expression modes of the three *CBF*s were different and that the gene expression of *DlCBF1/2* was induced by a cold signal in longan, whereas that of *DlCBF3* was not.

### Subcellular Localization of Longan CBF Proteins

**Figure 3:**
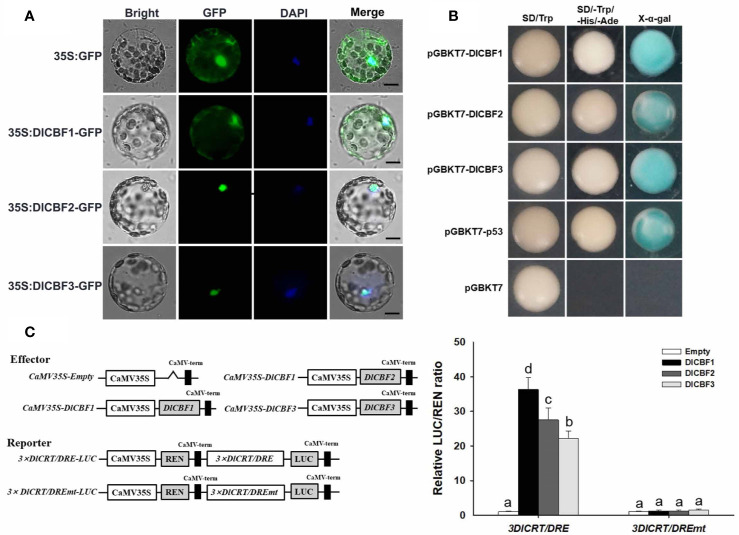
Subcellular localization and transactivation assay of DlCBF1/2/3. **(A)** Subcellular localization of 35S:DlCBF1/2/3-GFP in *N. benthamiana* protoplasts. 35S: GFP was used as a negative control. BF, bright-field; GFP, GFP fluorescence; DAPI, nuclear localization (pseudo-color, blue); Merged, merged images of GFP and DAPI. Scale bar, 10 μm. **(B)** Transactivation assay of DlCBF1/2/3 in yeast cells. pGBKT7-DlCBF1/2/3 fusion plasmids, pGBKT7-p53 (positive control) and pGBKT7 (negative control) were transformed into the Y2HGold yeast strain and cultured on SD/-Trp and SD/-Trp-His-Ade selective media. The yeast cells plated on SD/-Trp-His-Ade were then stained with X-α-gal. **(C)** CRT/DRE-binding specificity assay of DlCBFs. The effector of the dual-luciferase reporter system contained DlCBFs, the reporter harboring 3× DlCRT/DRE sequences (GCCGACAGG) or 3× DlCRT/DRE mutant sequences (GAATCAAGG). Effector and reporter cotransformation of *N. benthamiana* leaves for transient transformation. LUC and REN luciferase activities in leaves were measured, and the CRT/DRE-binding activities of DlCBFs were calculated by the ratio of LUC to REN. The error bars indicate the SDs from six biological replicates. The different letters above the bars indicate significant differences at the P < 0.05 level according to Duncan's multiple comparison tests.

Sequence analysis showed that all DlCBF1/2/3 contained an NLS region. To verify the subcellular localization of DlCBF1/2/3, a 35S:DlCBF1/2/3-GFP fusion construct and 35S: GFP empty vector were transiently expressed in leaf epidermal cells of *N. benthamiana*. In the leaf protoplasts, the fluorescence signals of 35S:DlCBF2/3-GFP were detected exclusively in the nucleus, coincident with the fluorescence signal of DAPI staining in the nucleus. The fluorescence signal of 35S:DlCBF1-GFP was detected in the nucleus as well as in the cytoplasm (**Figure 3A** and **Supplementary Figure 2**).

### Transcriptional Activation of DlCBF1/2/3 in Yeast

Transcriptional activation is a basic function of TFs. In this study, the Y2H Gold yeast system was used to analyze the transcriptional activation activity of DlCBF1/2/3. All the Y2H Gold yeast strains transfected with pGBKT7-DlCBF1/2/3, the pGBKT7-53+pGADT7-T positive control, or the pGBKT7 negative control grew normally on SD/-Trp media, indicating that the exogenous plasmids were successfully transferred into yeast strains. Furthermore, the yeast cells containing pGBKT7-DlCBF1/2/3 and the positive control grew well on SD/-Leu/-Trp/-Ade media and presented strong α-galactosidase activity, whereas the negative control did not grow on SD/-Leu/-Trp/-Ade media (**Figure 3B**). These results imply that DlCBFs have transcriptional activity in yeast.

### Specificity Binding Analysis of DlCBF1/2/3 With the CRT/DRE Motif

The CRT/DRE-binding specificity of DlCBFs was analyzed using a dual-luciferase reporter system. The ratio of LUC to REN of the pGreenII 62-SK empty vector was used as a negative control (the value was set as 1). When the effector containing DlCBFs interacted with the reporter harboring 3× DlCRT/DRE sequences, the ratio of LUC to REN was significantly higher than that of the negative control. When the effector containing DlCBFs interacted with the reporter harboring 3× DlCRT/DREmt sequences, the ratio of LUC to REN was not significantly different from that of the negative control (**Figure 3C**). These results indicate that DlCBF1/2/3 TFs could bind specifically to the longan CRT/DRE motif to activate the expression of the LUC reporter but could not bind to the mutant CRT/DRE motif.

### Overexpression of *DlCBF1/2/3* Enhances the Freezing Tolerance of Transgenic *Arabidopsis*

**Figure 4:**
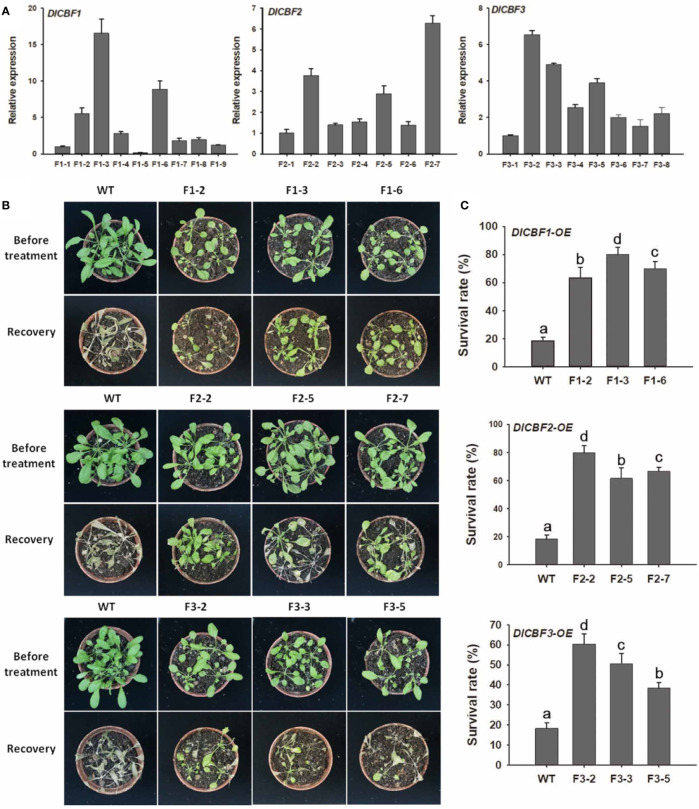
Overexpression of *DlCBF1/2/3* in Arabidopsis enhanced cold tolerance. **(A)** Confirmation of *DlCBF1/2/3* transcript levels in transgenic lines and WT plants. *AtACTIN2* was used as an internal control. **(B)** Phenotypes and **(C)** survival rates of the transgenic lines and WT plants after freezing. Three-week-old pot-grown Arabidopsis plants were treated with −4°C for 6 h, after which they were allowed to recover for 6 days; 20 seedlings per line were included in each freezing treatment. The error bars indicate the SDs from three biological replicates. The different letters above bars indicate significant differences at the *P* < 0.05 level according to Duncan's multiple comparison tests.

To further investigate the biological function of *DlCBF1/2/3* in cold tolerance, WT Arabidopsis Col-0 was transformed with plasmids harboring the *DlCBF1*-, *DlCBF2*- or *DlCBF3*-coding regions. Independent transgenic lines (T1 generation) were obtained based on kanamycin resistance selection and genomic PCR verification. Homozygous T3 lines were obtained on the basis of 3:1 segregation for kanamycin resistance. RT-qPCR was used to further determine the expression levels of the target genes in the homozygous transgenic lines. Three T3 transgenic lines presenting high expression of *DlCBF1* (F1-2, F1-3, and F1-6), *DlCBF2* (F2-2, F2-5, and F2-7) and *DlCBF3* (F3-2, F3-3, and F3-5) were ultimately selected for subsequently cold tolerance experiments (**Figure 4A**).

Compared with the WT plants, the transgenic Arabidopsis plants exhibited dwarf phenotypes, and the latter presented smaller, thicker, darker leaves; greater numbers of rosette leaves; shorter stems and inflorescences; and a later flowering time (**Supplementary Figure 3**). These phenotypes indicate that DlCBF1/2/3 affects the growth and development of Arabidopsis plants.

The transgenic lines and WT plants were subjected to a freezing treatment to determine the effects of *DlCBF1*, *DlCBF2*, and *DlCBF3* on freezing tolerance. For the freezing treatment, transgenic lines and WT plants displaying normal growth were selected for treatment of −4°C for 6 h, after which they were allowed to recover for 6 days. After the freezing stress, almost all WT plants died, while most of transgenic plants were still alive and resumed growing (**Figure 4B**). The survival rates of the *DlCBF1-OE*, *DlCBF2-OE*, and *DlCBF3-OE* transgenic lines were 63.3% to 80.2%, 61.7% to 79.8%, and 38.3% to 60%, respectively, which were significantly higher than those of the WT plants (17.5-19.3%). The survival rate of the *DlCBF3-OE* lines was lower than that of the *DlCBF1-OE* and *DlCBF2-OE* lines after freezing treatment, indicating that the freezing tolerance of the *DlCBF3-OE* lines was weaker than that of the *DlCBF1-OE* and *DlCBF2-OE* transgenic lines (**Figure 4C**). These results indicated that overexpression of *DlCBF1*, *DlCBF2*, and *DlCBF3* could improve the freezing resistance of plants. Moreover, compared with the *DlCBF3* gene, the *DlCBF1* and *DlCBF2* genes might play more important roles in regulating the cold resistance of longan.

### Overexpression of *DlCBF1/2/3* Alters Ion Leakage, Proline, MDA, and Reactive Oxygen Species Contents During Cold Stress

Physiological indexes such as ion leakage, proline levels, and MDA and reactive oxygen species (ROS) accumulation are used to assess the stress tolerance of plants in response to abiotic stress (Xu et al., 2014; Lu et al., 2017; Yuan et al., 2017). Ion leakage is a representative indicator of cell membrane damage during the plant response to abiotic stress. Proline enhances plant stress resistance by maintaining cellular osmotic balance and promoting normal membrane and protein biological functions. ROS, including H_2_O_2_, O_2_^.-^ and hydroxyl radicals (OH^-^), are considered to be inevitable products produced during plant responses to environmental stress (Tyystjarvi, 2013). Stress-induced ROS further break down polyunsaturated lipids, resulting in the production of MDA.

**Figure 5:**
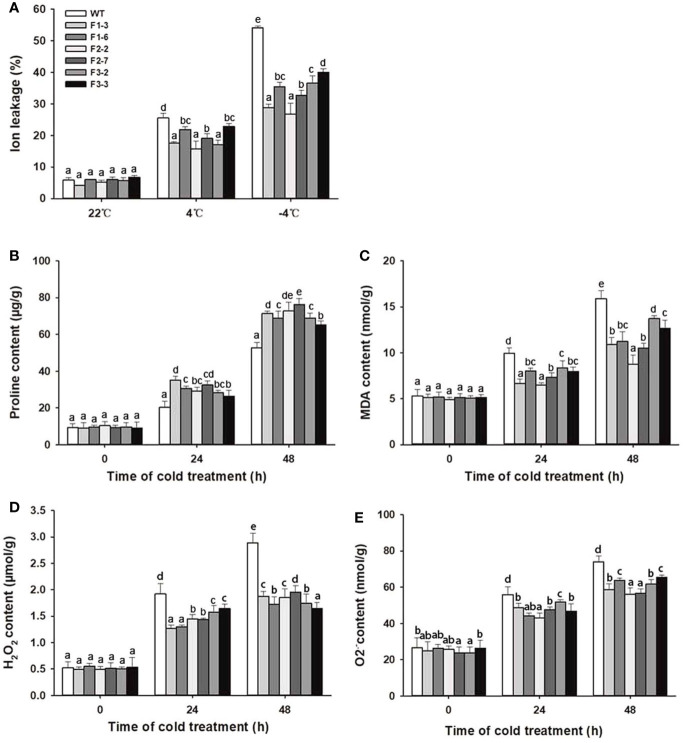
Change trends of physiological parameters of transgenic lines and WT plants under cold stress. **(A**–**E)** Ion leakage, proline, MDA, H_2_O_2_, and O_2_^.−^ contents were measured in 3-week-old transgenic lines and WT plants under cold stress. The error bars indicate the SDs from three biological replicates. The different letters above bars indicate significant differences at the *P* < 0.05 level according to Duncan's multiple comparison tests.

Under normal ambient temperature, there were no significant differences in the above physiological indexes between the *DlCBF1/2/3-OE* lines and the WT plants. After cold treatment, ion leakage, proline levels, and MDA and ROS contents gradually increased in both the transgenic lines and WT plants. Among these parameters, the ion leakage and MDA and ROS contents in the transgenic lines were distinctly lower than those in the WT plants at the peak value. In contrast, the proline level in the transgenic lines was significantly higher than that in the WT plants (**Figures 5A–E**). These results demonstrated that overexpression of *DlCBF1/2/3* could enhance the cold resistance by increasing proline accumulation, reducing ion leakage, and decreasing ROS and MDA production of transgenic plants under low-temperature stress.

### Overexpression of *DlCBF1/2/3* Affects the Expression of Endogenous Cold-Responsive Genes Under Cold Stress

**Figure 6:**
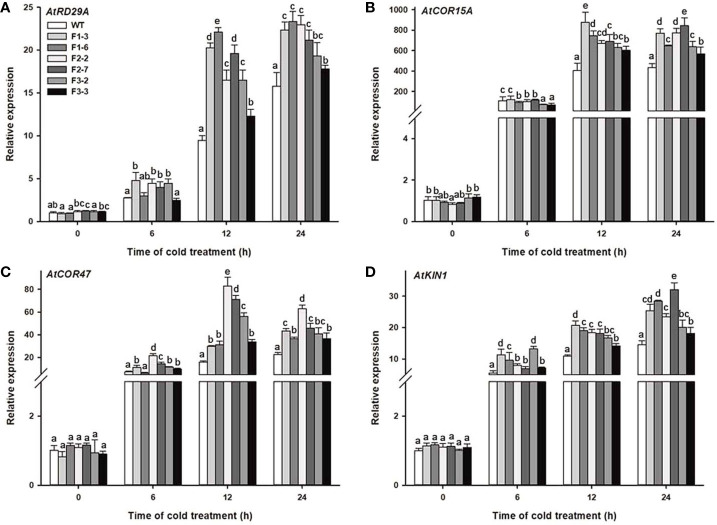
Expression levels of cold-responsive genes of transgenic lines and WT plants under cold treatment. **(A**–**D)** Changes in the expression of cold-responsive genes (*AtRD29A*, *AtCOR15A*, *AtCOR47*. and *AtKIN1*) were determined by qRT-PCR. Three-week-old transgenic lines and WT plants were treated with 4°C for 0, 6, 12, and 24 h. *AtACTIN2* was used as an internal control. The error bars indicate the SDs from three biological replicates. The different letters above the bars indicate significant differences at the *P* < 0.05 level according to Duncan's multiple comparison tests.

To further clarify the molecular mechanism of the cold response of the *DlCBF1/2/3*-OE transgenic lines, qRT-PCR was used to analyze the expression changes of cold-responsive genes in both the transgenic lines and WT plants under low-temperature stress. Previous reports have indicated that the promoters of cold-responsive genes contain one or more conserved CRT/DRE cis-elements, such as *AtRD29A*, *AtCOR47*, *AtCOR15A*, and *AtKIN1*, which are representative target genes regulated by CBF (Stockinger et al., 1997). Under normal growth conditions, the expression of COR genes in the transgenic lines and WT plants was relatively low. However, after 24 h of cold stress, the expression of the COR genes gradually increased to a peak value in both the transgenic lines and WT plants. Furthermore, the peak expression of COR genes in the transgenic lines was significantly higher than that in the WT plants (**Figures 6A–D**). The above results showed that overexpression of *DlCBF1/2/3* could positively regulate the expression of endogenous CBF downstream COR genes under cold stress, thus enhancing the cold resistance of transgenic plants.

## Discussion

As a southern subtropical fruit tree species, longan is highly sensitive to cold. Cold stress is one of the most important influencing factors restricting longan production in South China. However, little is known about the molecular mechanism underlying the response to cold stress in *D. longan*. Previous studies have revealed that CBF TFs play crucial roles in plant responses to cold stress (Liu et al., 2018). However, the function of CBF genes has not been studied in longan. Therefore, research on the CBF-dependent cold stress response signaling pathway in longan can provide new insight for breeding cold-resistant plants.

In this study, three CBF homologs named DlCBF1/2/3 were identified from longan, in which DlCBF1 and DlCBF2 contain a typical AP2 domain and two CBF characteristic conserved sequences, PKKP/PKKPAGR and DSAWR (Jaglo et al., 2001), which is similar to CBF proteins in Arabidopsis and in other plant species. Nevertheless, the CBF signature domains of DlCBF3 were not conserved, but these sequence differences had no significant impact on the subcellular localization, the specific binding to the CRT/DRE motif or the transcriptional activation of the DlCBF3 protein.

Genetic transformation analysis revealed that *DlCBF1/2/3* overexpression in Arabidopsis enhanced cold tolerance by regulating ion leakage and MDA and ROS accumulation and increased the expression of cold-responsive genes involved in the CBF cold response signaling pathway. The biological function of *DlCBF1/2* is similar to that of CBF orthologs from other plants species bearing fruit. Heterologous overexpression of the sweet cherry *PaCBF* and Vitis *VvCBF1/4* genes in Arabidopsis promotes cold tolerance in transgenic plants (Kitashiba et al., 2004; Siddiqua and Nassuth, 2011). Peach *PpCBF1* overexpressed in apple induced the expression of *MdCBF1/2*, which increased the anthocyanin content, thereby enhancing the cryotolerance of transgenic apple plants (Wisniewski et al., 2011). Additionally, overexpression of *DlCBF1/2/3* resulted in plant dwarfing and late flowering in transgenic Arabidopsis. Similar slow-development phenotypes were observed in response to overexpression of *AtCBF1* and orthologous genes from other woody plant species, such as *CsCBF* from tea plant (*Camellia sinensis*) and *PmhCBFc* from mei (*Prunus mume*) in Arabidopsis (Peng et al., 2016; Yin et al., 2016). These results imply that the biological functions of *DlCBF1/2/3* are conserved to some extent and are involved in the positive regulation of cold resistance in longan.

Intriguingly, expression analysis revealed that *DlCBF1/2* were expressed at relatively low levels in the apical buds and that their expression was induced by cold after 3 h and then peaked at 9 h after cold stress, which was consistent with the expression pattern of *MeCBF1* in cassava, a tropical crop species. *MeCBF1* was expressed at low levels in the apical buds, and the apical bud tissue of cassava was consistently more sensitive to cold stress (An et al., 2012). The expression of *MeCBF1* was induced after 4 h and peaked at 9 h under cold treatment (An et al., 2017). Similarly, expression of *HbCBF1* in rubber tree (*Hevea brasiliensis*), a tropical industrial plant species, was detected at 4 h and peaked at 8 h after cold stress (Cheng et al., 2015). However, cold-induced *AtCBF*s were expressed within 15 min, and their expression peaked within 3 h under cold treatment (Gilmour et al., 1998; Novillo et al., 2007). In addition, comparison of the 1-kb promoter sequences indicated that the *DlCBF1/2* promoter fragments contain multiple low temperature response cis-elements (LTR, CCGAAA) and MYC (CAACTG, CACATG, CACATG), while the *DlCBF3* promoter fragment does not contain LTR cis-elements but contains multiple MYB (TAACTG) cis-elements, which are related to drought and the heat stress response (Urao et al., 1996) (**Supplementary Table 5**). The results of our study are similar to those of a *MeCBF1* promoter analysis in cassava (An et al., 2017). The differences in these promoter elements may be responsible for the differences in the expression patterns of *CBF* genes of longan, but further study is required.

The expression of the *DlCBF3* gene was not induced by low temperature and was expressed at low levels in the tissues tested in this study, and the survival rate of the *DlCBF3-OE* lines was lower than that of the *DlCBF1-OE* and *DlCBF2-OE* lines after freezing treatment. These results indicated that *DlCBF1/2* might play a more important role than *DlCBF3* in the cold resistance of longan. Previous reports have shown that mutations in the PKKP/RAGR sequences with in the NLS motif influence the ability of AtCBF1 to bind to the promoter of the *COR15A* gene and reduce its expression under cold conditions (Canella et al., 2010). In Arabidopsis populations growing in warm climatic zones, a base mutation in *AtCBF2* inhibits the transcriptional activation of the AtCBF2 protein, thus attenuating *COR* gene expression (Kang et al., 2013; Gehan et al., 2015). Whether the sequence disparities of *DlCBF3* affect its ability to regulate the expression of downstream genes and reduce the cold resistance of longan warrants further in-depth study. In addition, longan is a cold-sensitive fruit tree species, and the reasons for the delayed expression of *DlCBF1/2* and the low induction of *DlCBF3* expression in response to cold stress remain unclear. Several fragment insertions are present within the *AtCBF3* promoter, leading to a decrease in *AtCBF3* expression to acclimate to warm temperatures (Kang et al., 2013; Gehan et al., 2015). ICE1 is one of the most important upstream regulators of *CBF* genes, and its homologue in longan, DlICE1, was verified to act as a positive regulator in the longan response to cold stress (Yang et al., 2019). Therefore, the characteristics of the promoters of *DlCBF1/2/3* and the mechanism by which DlICE1 regulates *DlCBF1/2/3* transcription need further study. Moreover, the upstream regulatory factors of *DlCBF1/2/3*, especially the negative regulatory factors, warrant further attention.

In summary, three novel CBF orthologs, *DlCBF1/2/3*, were identified from the subtropical fruit tree species *D. longan*. DlCBF1/2/3 are mainly localized in the nucleus and specifically bind to the CRT/DRE motif. Although overexpression of *DlCBF1/2/3* in Arabidopsis enhanced the cold tolerance of transgenic plants, the expression of *DlCBF1/2* was low and slowed in the apical buds during cold stress, while *DlCBF3* barely responded to cold signals. Therefore, it is proposed that insufficient expression of *DlCBF1/2/3* during the longan cold stress response might be partially responsible for the cold sensitivity of longan.

## Data Availability Statement

Publicly available datasets were analyzed in this study. This data can be found here: MN504651, MN504652, MN504653 (NCBI database).

## Author Contributions

XY, CL, and JF conceived the research and designed the experiments. XY, RW, HJ, and QC performed the experiments and analyzed the data. QC, XB, JZ, and GH were involved in the data analysis. XY mainly wrote the manuscript. CL and JF revised and approved the manuscript.

## Funding

This study was supported by the Key-Area Research and Development Program of Guangdong Province, China (2020B020220006), the Science and Technology Planning Project of Guangdong Province, China (2017B020201011, 2016A020210096, and 2015B020202010), the Science and Technology Program of Guangzhou, China (201604020188), the Longan Innovation Team of Modern Agricultural Industrial Technology Systems of Guangdong Province, China (2020KJ123).

## Conflict of Interest

The authors declare that the research was conducted in the absence of any commercial or financial relationships that could be construed as a potential conflict of interest.
