# Supplementary material for: Three Novel C-Repeat Binding Factor Genes of Dimocarpus longan Regulate Cold Stress Response in Arabidopsis
Source: Front Plant Sci. 2020 Jul 7;11:1026. doi: 10.3389/fpls.2020.01026 (PMC7358405; doi:10.3389/fpls.2020.01026)
Supplement: Supplementary file 1 [file DataSheet_1.docx]

Supplementary Material

**Supplementary Table 1. Primers used for the cloning of *DlCBF1/2/3* and RT-qPCR.**

| Primer name | Forward primer (5’ to 3’) | Reverse primer (5’ to 3’) |
| --- | --- | --- |
| *DlCBF1* | ATGGAGTTTTTGTCGAGCTA | TTATATTGAGTAACTCCACA |
| *DlCBF2* | ATGGACATGTTAAGTTATCT | TCAAAATGAAAAACTCCACA |
| *DlCBF3* | ATGGAACATAAAGATGAGTA | TCAGTCACTCCACAAAGTCA |
| *QDlCBF1* | GAAGCGAAGGACATACAGAAGG | ACAGTCCAGGCATCCCAAA |
| *QDlCBF2* | CCCAAGGCTTCTCCTGATC | TGGTATGGTCATCATCTCCC |
| *QDlCBF3* | ATTCTCACACCACCAGCCAT | GTCACTCCACAAAGTCAAGTCT |
| *DlcACTIN* | TTCCGTTGCCCAGAAGTCCTCTTC | CTTGCTCATACGGTCGGCAATACC |
| *QAtRD29* | GCCGAGAAACTTCAGATTGG | CCATTCCTCCTCCTCCTTTC |
| *QAtCOR15A* | GCAGATGGTGAGAAAGCGAA | GGCATCCTTAGCCTCTCCTG |
| *QAtCOR47* | CAGTGTCGGAGAGTGTGGTG | ACAGCTGGTGAATCCTCTGC |
| *QAtKIN1* | TGGAGCTGGAGCACAACA | GACCCGAATCGCTACTTGTTC |
| *AtACTIN2* | TAACAGGGAGAAGATGACTCAGATCA | AAGATCAAGACGAAGGATAGCATGAG |

Note: F, forward primer; R, reverse primer.

**Supplementary Table 2. Primers used for vector construction.**

| Primer name | Primer sequence (5’-3’) | RECS |
| --- | --- | --- |
| DlCBF1-GFP-F | GGTATCGAT**AAGCTT**ATGGAGTTTTTGTCGAGCTATTCTG | *Hind* Ⅲ |
| DlCBF1-GFP-R | CATACTAGT**GGATCC**TATTGAGTAACTCCACAGTGAC | *Bam*H I |
| DlCBF2-GFP-F | GGT ATCGAT**AAGCTT**ATGGACATGTTAAGTTATCTATCAG | *Hind* Ⅲ |
| DlCBF2-GFP-R | CATACTAGT**GGATCC**AAATGAAAAACTCCACAGTGAG | *Bam*H I |
| DlCBF3-GFP-F | GGTATCGAT**AAGCTT**ATGGAACATAAAGATGAGTACCAA | *Hind* Ⅲ |
| DlCBF3-GFP-R | CATACTAGT**GGATCC**GTCACTCCACAAAGTCAAGTCT | *Bam*H I |
| DlCBF1-BD-F | GCCATGGAGGCC**GAATTC**ATGGAGTTTTTGTCGAGCTA | *Eco*R I |
| DlCBF1-BD-R | CTGCAGGTCGAC**GGATCC** TTATATTGAGTAACTCCACA | *Bam*H I |
| DlCBF2-BD-F | GCCATGGAGGCC**GAATTC**ATGGACATGTTAAGTTATCT | *Eco*R I |
| DlCBF2-BD-R | CTGCAGGTCGAC**GGATCC**TCAAAATGAAAAACTCCACA | *Bam*H I |
| DlCBF3-BD-F | GCCATGGAGGCC**GAATTC**ATGGAACATAAAGATGAGTA | *Eco*R I |
| DlCBF3-BD-R | CTGCAGGTCGAC**GGATCC**TCAGTCACTCCACAAAGTCA | *Bam*H I |
| DlCBF1-OE- F | TCTACAACTACA**TCTAGA**ATGGAGTTTTTGTCGAGCTA | *Xba* I |
| DlCBF1-OE-R | CTTGCATGCCAAT**TCTAGA**TTATATTGAGTAACTCCACA | *Xba* I |
| DlCBF2-OE-F | TTCTACAACTACA**TCTAGA**ATGGACATGTTAAGTTATCT | *Xba* I |
| DlCBF2-OE-R | CTTGCATGCCAAT**TCTAGA**TCAAAATGAAAAACTCCACA | *Xba* I |
| DlCBF3-OE-F | TCTACAACTACA**TCTAGA**ATGGAACATAAAGATGAGTA | *Xba* I |
| DlCBF3-OE-R | CTTGCATGCCAAT**TCTAGA**TCAGTCACTCCACAAAGTCA | *Xba* I |
| p62SK-DlCBF1-F | TAGAACTAGT**GGATCC**ATGGAGTTTTTGTCGAGCTATTCTG | *Bam*H I |
| p62SK-DlCBF1-R | GCTTGATATC**GAATTC**TTATATTGAGTAACTCCACAGTGAC | *Eco*R I |
| p62SK-DlCBF2-F | TAGAACTAGT**GGATCC**ATGGACATGTTAAGTTATCTATCAG | *Bam*H I |
| p62SK-DlCBF2-R | GCTTGATATC**GAATTC**TCAAAATGAAAAACTCCACAGTGAG | *Eco*R I |
| p62SK-DlCBF3-F | TAGAACTAGT**GGATCC**ATGGAACATAAAGATGAGTACCAAT | *Bam*H I |
| p62SK-DlCBF3R | GCTTGATATC**GAATTC**TCAGTCACTCCACAAAGTCAAGTCT | *Eco*R I |
| 3DlCRT-LUC-F | ATAGGGCGAATTG**GGTACC**GGCCGACAGGGCCGACAGGGCCGACAG**GGATCC**ACTAGTTCTAGA | *Bam*HI/*Kpn* I |
| 3DlCRT-LUC-R | TCTAGAACTAGT**GGATCC**CTGTCGGCCCTGTCGGCCCTGTCGGCC**GGTACC**CAATTCGCCCTA | *Bam*H I/*Kpn* I |
| 3DlCRTmt-LUC-F | ATAGGGCGAATTG**GGTACC**GGAATCAAGGGATATCAGGGATATCAG**GGATCC**ACTAGTTCTAGA | *Bam*H I/*Kpn* I |
| 3DlCRTmt-LUC-R | TCTAGAACTAGT**GGATCC**CTGATATCCCTGATATCCCTTGATTCC**GGTACC**CAATTCGCCCTA | *Bam*H I/*Kpn* I |

Note: F, forward primer; R, reverse primer; RECS, [restriction](javascript:;) [enzyme](javascript:;) [cutting](javascript:;) [site](javascript:;); sequences homologous to those the vectors are underlined.

**Supplementary Table 3. Information and characteristics of longan *CBF* genes.**

| Gene name | ID | Gene location | ORF (bp) | No. aa | MW (kDa) | pI |
| --- | --- | --- | --- | --- | --- | --- |
| *DlCBF1* | Dlo_007317.1 | scaffold158:270499-271185 (+) | 687 | 228 | 25.13 | 5.21 |
| *DlCBF2* | Dlo_005616.1 | scaffold14:1145821-1146546 (-) | 726 | 241 | 26.77 | 6.24 |
| *DlCBF3* | Dlo_028757.1 | scaffold700:121825-122460 (+) | 636 | 211 | 23.71 | 5.60 |

Note: aa, amino acids; MW, [molecular weight](javascript:;); pI, [isoelectric](javascript:;) point.

**Supplementary Table 4. Identity matrix of nucleotide/amino acid sequences of *DlCBF1/2/3*.**

| Identity of nucleotide sequences (%) | Score of identity for amino acid sequences (%) | | |
| --- | --- | --- | --- |
|  | DlCBF1 | DlCBF2 | DlCBF3 |
| DlCBF1 | — | 54.73^a^ | 47.41^a^ |
| DlCBF2 | 58.88^b^ | — | 38.59^a^ |
| DlCBF3 | 50.22^b^ | 46.31^b^ | — |

Note: a, identity of amino acid sequences; b, identity of nucleotide sequences.

| **Site Name** | **Sequence** | ***DlCBF1*** | ***DlCBF2*** | ***DlCBF3*** | **Function** |
| --- | --- | --- | --- | --- | --- |
| ABRE | CACGT, ACGTGGC,  CANNTG | 3 | 2 | 4 | abscisic acid responsiveness |
| ARE | TGGTTT | 1 | 0 | 1 | essential for the anaerobic induction |
| CGTCA-motif | CGTCA | 1 | 2 | 1 | MeJA-responsiveness |
| circadian | CAANNNNATC | 0 | 1 | 0 | circadian control |
| G-Box | CACGTT, CACGTG, | 0 | 3 | 3 | involved in light responsiveness |
| ERE | ATTTCAAA | 1 | 1 | 1 | ethylene-responsive element |
| HSE | AGAAAATTCG | 1 | 0 | 0 | heat stress responsiveness |
| LTR | CCGAAA | 2 | 1 | 0 | low-temperature responsiveness |
| MYB | TAACTG | 0 | 1 | 4 | involved in drought-inducibility |
| MYC | CAACTG, CACATG,  CACATG | 2 | 1 | 3 | MYC binding site |
| TCA-element | CCATCTTTTT | 0 | 0 | 1 | salicylic acid responsiveness |
| W box | TTGACC | 0 | 1 | 1 | WRKY binding site |
| WUN-motif | AAATTTCTT | 0 | 1 | 1 | wound-responsive element |

**Supplementary Table 5. Promoter sequence comparison analysis of *DlCBF1/2/3*.**


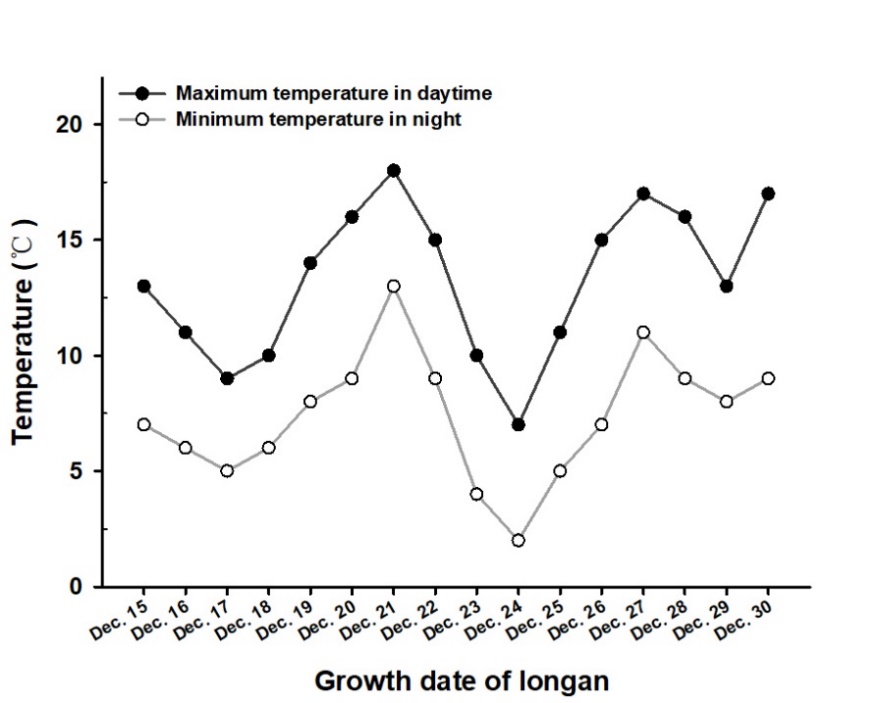


**Supplementary Figure 1．**Temperature fluctuations during longan development in late December 2016 in Guangzhou (23.12°N, 113.35°E)


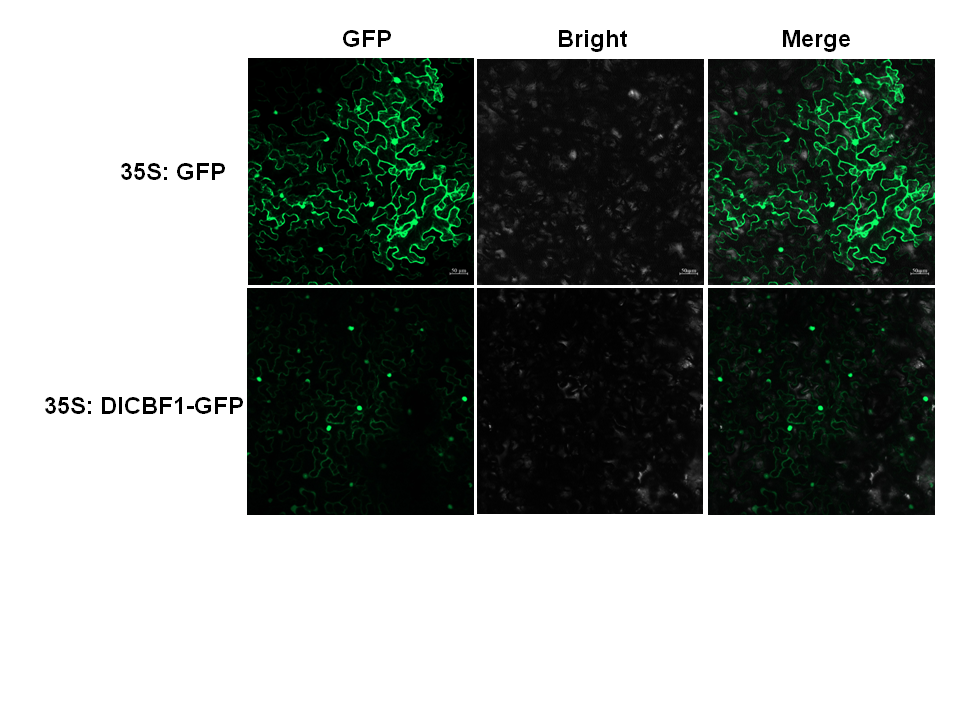


**Supplementary Figure 2**．Subcellular localization of DlCBF1 in *N. benthamiana* leaf epidermal cells. 35S:DlCBF1-GFP fusion constructs in *A. tumefaciens* strain GV3101 (psoup-p19) were transiently transformed into epidermal cells of *N. benthamiana* leaves. The GFP signals were visualized after 48 h of incubation at 25°C; 35S: GFP was used as a control. Bar=50 μm.


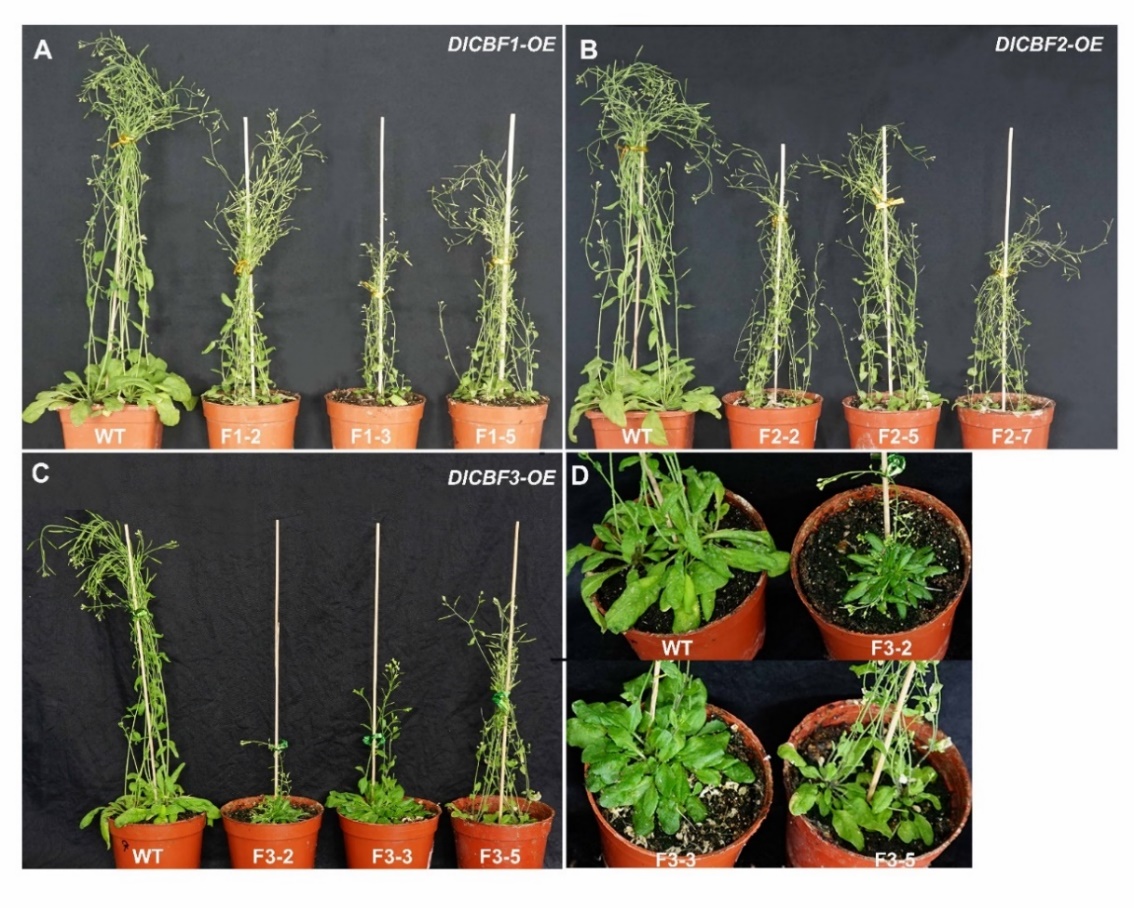


# Supplementary Figure 3. Phenotypes of *DlCBF1/2/3-OE* Arabidopsis plants. Six-week-old WT Arabidopsis Col-0 plants and *DlCBF1-OE* (A), *DlCBF2-OE* (B) and *DlCBF3-OE* (C) transgenic lines. (D) Top-down view of WT plants and *DlCBF3-OE* transgenic lines.
